# The Healing Hearts Together Randomized Controlled Trial and the COVID-19 Pandemic: A Tutorial for Transitioning From an In-Person to a Web-Based Intervention

**DOI:** 10.2196/25502

**Published:** 2021-04-06

**Authors:** Kathleen Lalande, Paul S Greenman, Karen Bouchard, Susan M Johnson, Heather Tulloch

**Affiliations:** 1 Division of Cardiac Prevention and Rehabilitation University of Ottawa Heart Institute Ottawa, ON Canada; 2 Université du Québec en Outaouais Gatineau, QC Canada; 3 Institut du Savoir Montfort Ottawa, ON Canada; 4 International Centre for Excellence in Emotionally Focused Therapy Ottawa, ON Canada

**Keywords:** web-based intervention, internet-based intervention, randomized controlled trial, COVID-19, research, tutorial, digital medicine, behavioral medicine, telehealth, telemedicine, cardiovascular rehabilitation

## Abstract

Supportive couple relationships are associated with reduced risk of chronic illness development, such as cardiovascular disease, as well as improved secondary prevention. Healing Hearts Together (HHT) is an 8-week couples-based intervention designed to improve relationship quality, mental health, quality of life, and cardiovascular health among couples in which one partner has experienced a cardiac event. A randomized controlled trial began in October 2019 to test the efficacy of the in-person, group-based HHT program as compared to usual care. In March of 2020, all recruitment, assessments, and interventions halted due to the COVID-19 pandemic. Guided by optimal virtual care principles, as well as by Hom and colleagues’ four-stage framework—consultation, adaptation, pilot-testing, and test launch—this paper is a tutorial for the step-by-step transition planning and implementation of a clinical research intervention from an in-person to a web-based format, using the HHT program as an example. Clinical and research considerations are reviewed, including (1) privacy, (2) therapeutic aspects of the intervention, (3) group cohesion, (4) research ethics, (5) participant recruitment, (6) assessment measures, (7) data collection, and (8) data analyses. This tutorial can assist clinical researchers in transitioning their research programs to a web-based format during the pandemic and beyond.

## Introduction

This tutorial aims to help clinical researchers transition their in-person research programs to a web-based format during the pandemic and beyond. To our knowledge, no literature exists on how to navigate the steps required to transition a randomized controlled trial (RCT), designed for in-person delivery, to an online world. No one has outlined, for example, contacts with sponsors and research ethics boards, recruitment procedures, or informed consent changes. This paper expands the previous transitional work in a clinical-only environment by accounting for the unique demands of clinical research and RCTs. The Healing Hearts Together (HHT) RCT provides researchers a current example of this challenging transition. We begin by briefly describing the original in-person HHT intervention and research protocol. We then guide clinical researchers through five important stages of transition: (1) consultation and assessment of needs, (2) adaptation of procedures and materials for web-based delivery, (3) adaptation of procedures and materials for the research protocol, (4) pilot sessions, and (5) the final launch of the web-based intervention and research protocol. We have also included a useful checklist of all the critical clinical and research elements that must be addressed so that the transition can be done in an efficient and ethical manner (see Multimedia Appendix 1 for checklist).

## Original In-Person HHT Program

A healthy couple relationship (ie, one in which partners feel loved, emotionally supported, respected, and cared for) is a significant protective factor, particularly against cardiac disease incidence and outcomes [1-5]. Healthy relationships can influence positive cardiac outcomes in direct and indirect ways. Direct examples include improved high-frequency heart rate variability and decreased diastolic blood pressure in supportive relationships [6-8]. Indirect links to cardiovascular health include adaptive behavioral pathways, such as partners influencing health behaviors (eg, prepare low sodium meals), modeling healthy behavior (eg, exercise), or assisting in the management of disease (eg, medication management) [9-11]. In contrast to their more distressed peers, happily married cardiac patients demonstrate stronger adherence to blood pressure medication regimens and cardiac rehabilitation programs [12,13].

Unfortunately, the reverse is also true. Relationship distress can have a negative impact on heart disease. A recent meta-analysis showed that poor social relationships were associated with a 29% increase in risk of incident coronary artery disease [14]. Uchino and colleagues found that coronary artery calcification scores were higher for individuals who expressed ambivalence rather than positivity about their couple relationship [15]. Other researchers have highlighted the indirect pathways by which relationship distress influences cardiac outcomes, including smoking and alcohol use in response to relationship problems [16].

Despite burgeoning evidence indicating that healthy relationships are vital for reducing chronic disease incidence and management, current secondary prevention programming inadvertently neglects a crucial resource for disease management: the patient’s partner. In order to address this gap in clinical care, the HHT program [17] was created with the aim of helping both cardiac patients and their partners better manage cardiac disease by strengthening the emotional bond between them. The goals of HHT are to help couples improve their relationship quality, mental health, quality of life, and cardiovascular health. The HHT couples-based intervention is adapted from the Hold Me Tight: Conversations for Connection (HMT) program [18], an intervention based on emotionally focused therapy (EFT) for couples, which is an empirically supported treatment for relationship distress [19,20]. EFT, HMT, and HHT are based on attachment theory, which states that humans have an innate need for close emotional bonds to significant others [10,11]. This need becomes especially pertinent when faced with a threat or stress (eg, a cardiac event); it triggers proximity seeking to “attachment figures” in order to regulate affect. EFT interventions help couples identify and articulate their vulnerability (eg, “I almost died/I almost lost you”) and respond to these feelings in comforting ways, thereby solidifying their sense of security and emotional connection [19,21]. Couples who achieve this connection are happier in their relationships and are more effective problem solvers (eg, managing cardiovascular disease) [22-24]. Inspired by HMT, the HHT program guides couples through seven conversations, based on EFT principles, in which they learn to clearly communicate their need for connection and reassurance, with a focus on heart disease and healthy coping.

Preliminary results from a proof-of-concept study indicated that couples who participated in the HHT program reported significant improvements in relationship quality, mental health, and quality of life [25]. Based on these promising results and to assess the efficacy of the HHT program on a larger sample with additional cardiovascular outcomes, an RCT was initiated. Eligible patients and their partners who consent to participation undergo a baseline assessment and then are randomly assigned to either the HHT program or usual care at the hospital. Usual care participants are followed by their physician for clinical assessment and are referred to the standard programming at the center. All participants are reassessed at 8 and 24 weeks.

Prior to the COVID-19 pandemic, participants who were randomly assigned to the HHT group attended, in person, eight weekly 2-hour sessions led by two facilitators. The eight sessions focus on understanding love, attachment, and their relationship to heart health; provide an opportunity to share experiences related to cardiovascular disease with partners and peers; and assist in identifying and improving communication patterns that may inhibit positive interactions and healthy behaviors. Throughout each 2-hour session, participants are introduced to concepts through didactic presentations, videos, group and couple discussions, and homework exercises. Additional study procedures and intervention content will be reviewed below as the in-person and web-based HHT programs are compared.

## Stage I: Consultation and Assessment of Needs for the Intervention and Research Protocol

### Overview

**Figure 1:**
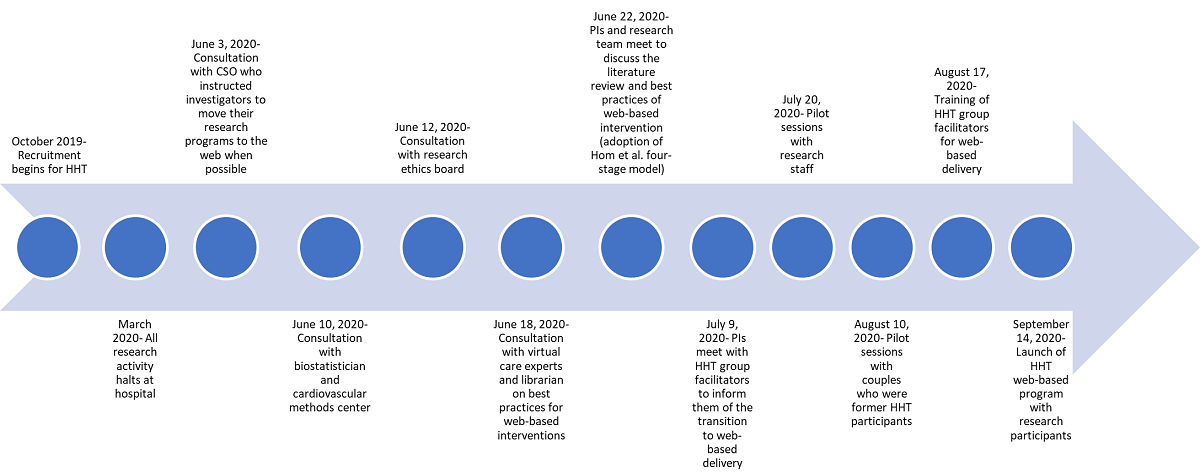
Timeline of the transition to a web-based intervention during COVID-19. CSO: chief scientific officer; HHT: Healing Hearts Together; PI: principal investigator.

During the first few months of the COVID-19 lockdown in March, April, and May of 2020, the chief scientific officer stopped all research in the hospital. At that time, it was not clear that HHT would have to transition to a web-based platform; many researchers were hoping that the situation with COVID-19 would stabilize within a few months. However, in June 2020, researchers at the hospital were asked to adapt their research protocols to allow for as much virtual care as possible. Following these directives, the HHT research team began to focus on how to do this while following best practices and preserving the integrity of the trial (see Figure 1 for a timeline of the transition to a web-based intervention during COVID-19).

### Literature Review

The principal investigators of HHT met at the beginning of June 2020 to outline a plan for transitioning to a web-based intervention as well as to review all study procedures to identify areas of required revision (eg, recruitment procedures and presentation of intervention content online). An examination of the current literature to aid in this transition was completed. We were able to identify best practices for virtual care (1) by seeking the help of a research librarian, who assisted with a literature review of resources on how to transition from an in-person psychological intervention to a web-based format, and (2) by consulting with internationally renowned experts in virtual care, who also directed our attention to pertinent literature.

The literature review yielded a small number of resources that provided a framework for the transition [26-33]. More specifically, these articles highlighted key areas of telehealth development, including patient involvement [29], technological support and training for the group facilitators [27], a strong working alliance during web-based therapy [33], ethical considerations [30], and the importance of comprehensive information technology (IT) training and support [28]. A systematic review of telehealth interventions delivering home-based support with group videoconferencing highlighted several benefits of web-based interventions, including improved accessibility, engagement with others facing similar challenges (eg, cardiovascular disease), development of health knowledge and skills, and improved mental health outcomes similar to face-to-face groups [28]. Finally, Hom and colleagues [26] provided a useful framework for modifying a clinical psychiatric program for a web-based platform using four stages: consultation, adaptation, pilot-testing, and test launch.

### Research Staff Communication

Following the literature review and consultation with virtual care experts, a focus on ensuring open and clear communication among the members of the research team, who were all working from home, was needed. We chose a web-based collaborative workspace (eg, Trello and Slack) to streamline staff communication and to assign tasks and deadlines to research staff. This avoided the hassle and confusion of sending multiple emails regarding project updates. Instead, we simply log in to our web-based communication board to view the task list to be completed and by whom. Regular weekly lab meetings were also transitioned to a videoconferencing platform. These meetings assisted in the organization of deliverables and facilitated lab staff cohesion, morale, and motivation during these unprecedented times.

### Updating the Funding Agency and Ethics Board

Shortly after research was halted at the hospital, the study’s federal funding agency reached out to the nominated principal investigator proactively with an update reassuring all researchers that the funding window would be extended due to the COVID-19 crisis, and that no extra report or documentation would be required. The research coordinator updated the study registry, ClinicalTrials.gov, and the hospital’s electronic medical record, noting that the study was no longer actively recruiting. Researchers may need to contact their sponsors or funding agencies to inquire about their specific requirements.

Consultation with our institutional ethics board regarding required study updates for the transition from a predominantly in-person-based study to one with virtual methods was then conducted. Within days of the shutdown, we informed the research ethics board of all changes in research activities (eg, phone contact to inform patients, no recruitment, and temporary follow-up by mail). Although the move to a web-based HHT program does lower the risk of COVID-19 transmission to our participants, there is also an increased risk of privacy breaches with an online format. In response to privacy concerns, a form outlining the changes to our methods and protocol, noting strategies to protect personal health information and privacy, was submitted to the research ethics board for approval. Once the approval was received, a form requesting permission to restart our research study was submitted to our research services department lead by the chief scientific officer of our institution. In this form, we outlined the safety protocols for the in-person baseline and follow-up assessments at the hospital, including appropriate personal protective equipment, cleaning the workspace once participants have left, physical distancing when possible, and equipment changes to ensure that COVID-19 is not transmitted (eg, new valve in the carbon monoxide monitor). Our research staff attended a consenting and documentation seminar for studies conducting virtual recruitment, consent, and interventions at our facility. The principal investigator completed an online training seminar on how to use the hospital-approved secure videoconferencing platform linked with the hospital’s secure electronic medical record software. This information was subsequently shared with staff.

## Stage II: Adaptation of Procedures and Materials for Web-Based Delivery

### Optimal Web-Based Care Principles

To ensure that we were following best practices in the provision of psychological services via telepsychology, we referred to the guidelines provided by the American Psychological Association [34], the Canadian Psychological Association, and the Ontario Psychological Association, in addition to the previously mentioned resources from the literature review [26-32]. For the purposes of this tutorial, we will highlight the best practices that were most relevant to our study and the adjustments that were required for the research protocol.

### Provincial Jurisdiction of Psychological Services

Patients from the neighboring province of Quebec frequently seek services at our Ontario hospital. Because Quebec patients would no longer be able to come to the hospital to participate in the HHT program, the principal investigator contacted the Order of Psychologists of Quebec to obtain their permission to provide services to Quebec residents via a web-based platform. Due to the unusual pandemic circumstances, this permission was swiftly granted. It is recommended that clinician researchers ensure they have the appropriate credentials to provide care in the jurisdiction of the patient.

### Security and Transmission of Data and Information

As per the directives at our institution, the hospital-approved videoconferencing platform was employed to run the HHT groups online. The principal investigators reviewed every session of the HHT program and determined that the intervention elements could be delivered securely via videoconferencing.

### Appropriate Medium of Delivery

Another crucial factor in successful web-based therapy is the existence of adequate technology, equipment, and usability for research staff and participants. Previous research has shown that web-based interventions face common IT and visual issues, such as audio difficulties, delays, dropouts, background noise, difficulties downloading software, and poor lighting [28]. In response to these common challenges, a checklist for facilitators and potential participants was created to confirm that they had the necessary equipment, technology, and space to do HHT in a web-based format in a secure and private manner. It includes questions that assess whether they have (1) internet access (ie, Wi-Fi or ethernet) at home; (2) two devices that allow for videoconferencing (ie, camera and microphone), such as a desktop computer or laptop, a tablet or iPad, a Chromebook, or a smartphone; and (3) a quiet and private room in their residence.

## Stage III: Adaptation of Procedures and Materials for the Research Protocol

### Recruitment

Before the pandemic, recruitment was conducted in person at the hospital when patients came to see their cardiologists for follow-up appointments. Members of the research staff spoke with patients after their appointments and presented an overview of the HHT program. Interested patients agreed to be contacted by study staff, who described the study in greater detail and answered any questions. If still interested, an initial assessment appointment was scheduled. With the new COVID-19 restrictions, most clinical care is done virtually (ie, phone or video appointments). A new standard operating procedure for HHT recruitment was developed outlining the steps to call patients who have already given general consent to be contacted for research purposes at our center. With no in-person clinical visits, recruitment is now completed entirely over the phone. Members of the research staff call potential participants shortly after their phone consultation with their cardiologist and review the technology checklist before proceeding with the recruitment script. If patients do not have the necessary computer equipment, they are not eligible for the study at this time. As the pandemic restrictions ease, patients without the necessary technology will be provided the option to engage in person if randomized to the HHT condition.

### Informed Consent

As our study is nonregulated and low risk, our institutional ethics review board informed us that we could adopt a well-documented verbal consent process that permits the research coordinator to read the full written informed consent form to potential participants via telephone. Participants can then ask the research coordinator any questions they have about the study before granting verbal consent. The research coordinator must document the consent process in the participant’s research file and electronic medical record, with the help of a verbal consent checklist. If requested by the participant, the consent process may also be completed in person at the time of the assessment at the hospital. In this case, the in-person consent process will still be completed verbally with no transfer of paper between staff and participants.

Other secure methods of obtaining consent via electronic signature software and electronic consent platforms (eg, Research Electronic Data Capture [REDCap] e-consent framework, DocuSign, and Adobe Sign) may also be considered. The advantage of these systems is that they provide a user-friendly option for individuals to personally sign the consent form. However, there are potential disadvantages that include cost, the need for additional institutional approval, and complexity barriers for less tech-savvy individuals. Finally, there is the traditional method of obtaining informed consent by which the study team establishes a process, in alignment with research regulations, whereby the paper consent form is mailed to the participant and, following the consent discussion, the participant signs the paper consent form and mails it back to the institution. Researchers who are working with external sponsors need to verify with their institution and their sponsor the appropriate informed consent process to use during the pandemic.

### Assessment

In the original protocol, patients are asked to come to the hospital for a baseline assessment and two follow-up assessments. Physiological measurements are taken, including heart rate variability, blood pressure, height, weight, waist circumference, carbon monoxide concentration, and salivary samples. In addition, all the couples are asked to participate in a conflict resolution task (ie, a discussion of topics that engender conflict, such as finances, in-laws, or jealousy) that is video-recorded for analysis. The research team initially explored ways to complete these measures remotely, but realized it was not possible for many reasons (eg, cost, coordination of assessment, and variability in measurement). To facilitate coordination of staff on site, the hospital research support office implemented a shared calendar for staff to indicate when they would be on site so as to not exceed maximum pandemic capacity and to allow the coordination of clinical research activity among staff.

## Stage IV: Pilot-Testing and Streamlining of Procedures

### Pilot Tests

Once the HHT in-person intervention was adapted for web-based administration, the principal investigators (ie, trained group facilitators) ran pilot web-based HHT group sessions with volunteers from the research staff and their partners to identify any issues or problems. During these sessions, features of the videoconferencing platform were explored and problems addressed (eg, chat function and breakout rooms). During the traditional in-person format, the facilitators can quietly and respectfully check in with couples who are working privately on an in-class exercise together. The facilitators realized, however, that without the in-person visual cues, it would prove difficult to identify couples in need of assistance during the breakout sessions or to enter these sessions unannounced. Couples also have the option of notifying their hosts that they are in need of assistance. In addition, to prevent an intrusive interruption, facilitators now provide participants with a screen announcement—audio and video off—letting them know that a facilitator would like to join their breakout room. Next, permission to join is requested via audio, before their video image is shown. It is important to note that videoconferencing services now have excellent online support centers with helpful videos and tutorials where researchers can learn and explore the features and strategies that exist to enhance their web-based intervention. The lessons learned from the pilot tests were used to create separate tutorials for group facilitators and participants.

### Test Launch With Previous HHT Participants

After running the pilot sessions with volunteer research staff and their partners, the principal investigators conducted a pilot HHT session with couples who were previous participants of the in-person HHT program. The research coordinator contacted these individuals in advance to review the technology checklist and to provide a brief tutorial on how to use the videoconferencing platform. By consulting previous participants of the traditional in-person HHT group, the facilitators gained instructive feedback that contributed to treatment fidelity and acceptability of the web-based format [29]. In an informal postsession feedback conversation, the couples provided suggestions, including (1) visual format changes (ie, reduce the slide size) in order to make the presenter’s face more prominent to facilitate a personal connection, (2) offer participants a choice between receiving the printed session handouts by mail or digitally via email, and (3) purposefully enhance and monitor group cohesion. Methods to enhance group cohesion online have been suggested recently [35].

### Lessons Learned From the Pilot Sessions

**Figure 2:**
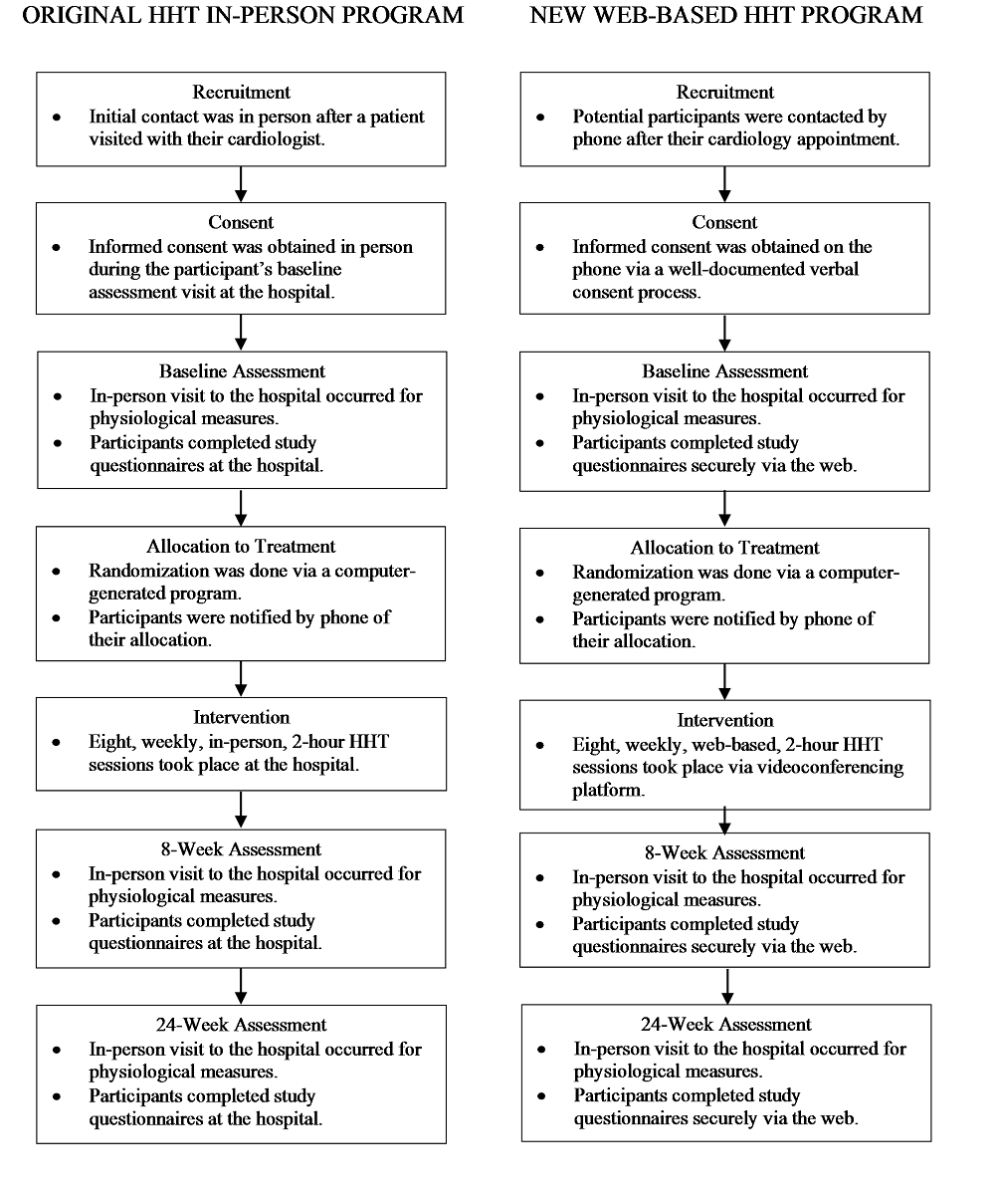
A comparison of the original in-person Healing Hearts Together (HHT) program and the web-based HHT program.

The most helpful step, of those outlined by Hom and colleagues [26], was pilot-testing the intervention to staff and previous HHT participants. Facilitators learned that to maximize group cohesion, maximize flow of conversation, and allow participants to contribute substantially to meaningful discussions, limiting the group to a maximum of 5 couples was required. During the sessions themselves, facilitators found it easier to manage the group dynamics by disabling the chat feature—except with the hosts—and by requesting that the participants use their mute function during the didactic and presentation portion of the session to minimize distraction and hearing difficulties. Finally, facilitators realized that it will be important to seek ongoing feedback from all the participants regarding IT issues or anything related to the web-based format; to that end, they employed a brief questionnaire for participants to fill out after the completion of the HHT program (see Multimedia Appendix 2 for the HHT Client Satisfaction Survey). With the pilot sessions completed, the new web-based HHT program was ready to launch (see Figure 2 for a comparison of the original in-person HHT program and the web-based HHT program).

## Stage V: Launch of the Web-Based HHT Intervention and Research Protocol

### Baseline Assessment

The baseline assessment visit is conducted at the hospital. Participants scheduled for the assessment are now screened for COVID-19 over the phone within 24 hours of their visit. Once they arrive at the hospital, they are rescreened at the hospital entrance and provided with a mask. Research staff wear appropriate personal protective equipment. Physical distancing regulations are respected, except when completing measurements such as heart rate variability, blood pressure, height, weight, waist circumference, carbon monoxide concentration, and saliva. Proper hygiene procedures are used at every stage of assessment. Questionnaires are completed by participants at home via a secure web application for building and managing web-based surveys and databases (eg, REDCap). If participants decline an on-site appointment, questionnaires can still be completed online and physiological measures are not completed.

### Allocation to Treatment

The randomization process has not changed with the new web-based format. Participants are randomized to the HHT program or to usual care using a computer-generated program at our cardiovascular methods center. Sequences are placed in sealed, numbered, opaque envelopes to ensure concealment of treatment allocation until after baseline data collection. The research coordinator allocates the next available number on study entry, logs all randomizations, and notifies participants of their allocated group immediately by phone. The research coordinator and participants are aware of group allocation. Research assistants, blinded to the participants’ treatment allocation, conduct both follow-up assessments.

### Intervention

The participants who are randomly assigned to the experimental intervention participate in the new web-based HHT group program. The research coordinator informs participants of their program start date, provides each couple with a brief tutorial of how to access the videoconferencing software, and sends them the intervention materials (eg, book and handouts) via email and/or post before the group start date. For extra technical support, participants receive an email with the prepared tip sheets to help them navigate the web-based format. The number (eight sessions) and duration (2 hours) of sessions remain the same in the web-based format. In this format, couples continue to benefit from the same didactic presentations, videos, and group and couple discussions that were offered in the traditional in-person format. After each web-based session, participants are asked to fill out a very brief feedback questionnaire.

### End-of-Treatment and Follow-Up Assessment

As with the baseline assessment, participants will be permitted to go in person to the hospital for their 8-week follow-up assessments. Nevertheless, it was necessary to create a contingency plan that allows for a more flexible approach. Should more strict hospital restrictions emerge, the research staff has prepared alternative means of gathering assessment data, (eg, recording the conflict resolution task via the videoconferencing platform, using self-reported unstandardized height and weight readings, and using self-reported blood pressure readings).

## CONSORT Reporting

The research coordinator updated the CONSORT data to include patients who were no longer eligible due to surpassing the eligibility window as well as patients who were unable to complete follow-up assessments due to the pandemic. In addition, revisions were made to the inclusion and exclusion criteria (eg, technology requirements and broader catchment area).

## Data Management and Analysis

As COVID-19 restrictions lift, a choice for the in-person versus the web-based HHT program may be provided to participants. However, this creates a new and potentially confounding variable that will need to be included in the database and explored in statistical analyses. A dichotomous variable indicating participation in the web-based or in-person HHT program will then be used as a covariate to control for the mode of delivery. Exploratory analyses can be used to investigate whether the delivery mode made a difference by using an interaction term between delivery mode and the intervention. Advice from the study statistician regarding the above procedures was sought. Researchers should consult with appropriate statistics advisors when interim analyses are conducted and changes to the data analysis plan are considered.

## Advantages of the New Web-Based Format

As we updated our protocol, several advantages of the web-based program became evident, both from a clinical and research perspective. These included the following:

1. Broader recruitment. With the necessity of weekly travel removed, the potential recruitment of patients living outside of a 1-hour radius from the hospital could now be included. Prior to the pandemic, participants who lived longer than an hour’s drive from the hospital were excluded, as it was seldom feasible for couples to drive an hour or more for eight weekly group sessions, in addition to the three assessment appointments, especially in the winter months. However, with the web-based format, participants are required to attend only three assessments on site. As such, from a research perspective, it is important to note that the reduced travel burden permits potential recruitment from rural areas, which will enrich the diversity of our sample.
2. Flexible scheduling. Flexibility in scheduling both participants and facilitators for the group sessions and assessments emerged as another benefit of the HHT web-based intervention. With no need to reserve group rooms or to arrange for parking at our busy hospital, finding mutually convenient times for the web-based sessions and for the in-person assessments has been optimized. In addition, all group facilitators can be trained online at their convenience individually or as a group by the research coordinator, a process that allows for streamlined, efficient training and ongoing support for the HHT facilitators [27].
3. Flexible methodology. For several years, the gold standard for the informed consent process has involved face-to-face interactions with potential participants. With the recent increase in web-based surveys and interventions, there has been more openness to online consent forms. However, hospital-based clinical trials have always favored the traditional in-person model for obtaining consent. The COVID-19 pandemic has required hospitals and research institutions to adopt more flexible means to obtain informed consent, while assuring high ethical standards. This flexibility toward informed consent and other aspects of methodology (eg, recruitment and web-based measures) will provide opportunities for researchers who would like to reach a broader population.

## Conclusions

When the principal investigators conceptualized and created the HHT program, they envisioned an in-person group program that would help couples grow closer and work well together to improve cardiac health. The COVID-19 pandemic forced hospitals to rethink the mode of delivery for their services. While these drastic changes undoubtedly prompt stress and invoke challenges, there are opportunities for clinical research to extend the reach of recruitment to hard-to-access populations (eg, patients with chronic conditions or mobility issues and patients who have family and work obligations that do not allow them to leave their homes) and, ultimately, promote patient-centered care. It is understood that researchers around the world will also have to take into consideration the subject matter of their research, as well as the cultural, environmental, occupational, and economic factors in their home countries. Despite the diverse nature of global research, this tutorial aims to serve as a brief, yet comprehensive, framework for clinical researchers facing the challenge to offer flexible and innovative web-based interventions.

## Appendix

Multimedia Appendix 1 Checklist for clinical researchers transitioning their research and intervention to a web-based platform.

Multimedia Appendix 2 Healing Hearts Together (HHT) Client Satisfaction Survey.

## Abbreviations

EFT: emotionally focused therapy
HHT: Healing Hearts Together
HMT: Hold Me Tight: Conversations for Connection
IT: information technology
RCT: randomized controlled trial
REDCap: Research Electronic Data Capture
