# Supplementary material for: The Healing Hearts Together Randomized Controlled Trial and the COVID-19 Pandemic: A Tutorial for Transitioning From an In-Person to a Web-Based Intervention
Source: J Med Internet Res. 2021 Apr 6;23(4):e25502. doi: 10.2196/25502 (PMC8025918; doi:10.2196/25502)
Supplement: Multimedia Appendix 1 [file jmir_v23i4e25502_app1.pdf]

Checklist for the transition to a web-based platform

- ☐ Consult with investigative team
- ☐ Contact sponsor
- ☐ Contact/update research ethics board
- ☐ Update clinical trials registry
- ☐ Choose a practical web-based collaborative workspace for team communication
- ☐ Literature review of best practices for web-based interventions
- ☐ Consider privacy/confidentiality issues
- ☐ Revise informed consent
- ☐ Revise protocol/intervention to reflect changes transitioning to web-based format
- ☐ Provide any necessary staff training for new web-based procedures
- ☐ Create and provide participant handouts to support the new web-based procedures
- ☐ Obtain institutional/ethics approval to use video-conferencing platform; purchase license
- ☐ Update/create standard operating procedures for the new recruitment methods
- ☐ Update/create standard operating procedures for the web-based intervention
- ☐ Contact/consult institutional research services/senior management for guidance/approval
- ☐ Follow institutional protocol and obtain approval before resuming recruitment/research
- ☐ Consult with statisticians/advisors regarding changes to data collection/analyses.
