# Supplementary material for: The Healing Hearts Together Randomized Controlled Trial and the COVID-19 Pandemic: A Tutorial for Transitioning From an In-Person to a Web-Based Intervention
Source: J Med Internet Res. 2021 Apr 6;23(4):e25502. doi: 10.2196/25502 (PMC8025918; doi:10.2196/25502)
Supplement: Multimedia Appendix 2 [file jmir_v23i4e25502_app2.pdf]

Hold Me Tight ® Healing Hearts Together (HHT) Satisfaction Survey

<u>Instructions:</u> Your thoughts and feedback are important to us and help us provide optimal services to our patients at the Heart Institute. Please take a few minutes to think about your experiences in group, and answer the following questions. We appreciate your honest and candid feedback.

1. Please indicate if you are a patient or spouse (please circle): ***patient*** ***spouse***

2. How do you describe yourself?

Man ***Woman*** ***Transgender*** ***Do not identify as a man, woman, or transgender***

3. Overall, how did you like the group?

1	2	3	4	5
Strongly Disliked	Disliked	Neutral	Liked	Strongly Liked

4. Please rate the program on the following dimensions, where 1 = poor and 5 = excellent:

Content	1	2	3	4	5
Facilitator interactions	1	2	3	4	5
Usefulness of session exercises	1	2	3	4	5
Usefulness of the homework	1	2	3	4	5
Content of video clips	1	2	3	4	5

5. Please rate each of the sessions, where 1 = poor and 5 = excellent:

Session 1: Understanding love and attachment	1	2	3	4	5
Session 2: The voices of patients and partners	1	2	3	4	5
Session 3: Recognizing demon dialogues	1	2	3	4	5
Session 4: Finding the raw spots	1	2	3	4	5
Session 5: Becoming open and responsive	1	2	3	4	5

Session 6: Forgiving injuries and trusting again	1	2	3	4	5
Session 7: Bonding Through Sex and touch	1	2	3	4	5
Session 8: Keeping your love alive	1	2	3	4	5

6. How many homework assignments did you complete (there were 7 in total)?

   - ☐ 0-2
   - ☐ 3-4
   - ☐ 5-6
   - ☐ All

7. During the course of this program did you learn something:

   About yourself? ***Yes*** ***No***
   About your partner? ***Yes*** ***No***
   About the impact that you have on one another? ***Yes*** ***No***

8. Please rate how you felt about the amount of discussion between group members. Please circle one:

   Not enough ***Just right*** ***Too much***

9. Has this HHT group changed your interactions with your partner regarding cardiac health management? If so, would you say your interactions are (please circle):

   worse ***the same*** ***better***

   Please describe.

____

____

____

____

____

10. Overall, did you get the outcomes that you were looking for?

   - ☐ Yes
   - ☐ No

Please explain.

11. What did you like most about this program?

12. What do you wish could have been added or done differently in this program?

13. Would you recommend this program to others?

- ☐ Yes
- ☐ No

Please tell us why?

14. Please use the space below for any additional comments for us.

If your Healing Hearts Together group sessions took place online, please complete the additional questions below.

The following questions are focused on your experience with the virtual platform.

15. Before the first session, were you concerned about experiencing difficulty when using technology?

- ☐ Yes
- ☐ No

16. Can you please elaborate on your concerns about difficulty using technology?

17. What type of device(s) did you use for your virtual group sessions?

- ☐ Desktop computer
- ☐ Laptop
- ☐ Tablet
- ☐ Smartphone video
- ☐ Smartphone voice only

18. Were there any problems with the following? Select the ones that apply.

- ☐ Quality of the audio (sound)
- ☐ Quality of the video (visual)
- ☐ Software set-up/Login process
- ☐ Scheduling process
- ☐ No problems

19. How much did the problem(s) impact your satisfaction with the virtual visit?

- ☐ The problem(s) did not impact my satisfaction with the virtual visit
- ☐ The problem(s) somewhat reduced my satisfaction
- ☐ The problem(s) greatly reduced my satisfaction

20. Did you need any technical help before or during your virtual visit?

- ☐ Yes
- ☐ No

21. Please add any comments about the technical help you required.

____

____

____

____

____

____

THANK YOU FOR TAKING THE TIME TO COMPLETE THIS SURVEY!
